# Understanding the bioeconomy through its instruments: standardizing sustainability, neoliberalizing bioeconomies?

**DOI:** 10.1007/s11625-022-01256-2

**Published:** 2023-01-06

**Authors:** Thomas Vogelpohl

**Affiliations:** 1 Institute of Sociology, Faculty of Social and Behavioural Sciences, Friedrich Schiller University Jena, Jena, Germany; 2 Present Address: Agricultural and Food Policy Group, Department of Agricultural Economics, Albrecht Daniel Thaer-Institute of Agricultural and Horticultural Sciences, Faculty of Life Sciences, Humboldt-Universität zu Berlin, Unter den Linden 6, 10099 Berlin, Germany

**Keywords:** Bioeconomy, Sustainability, Policy instruments, Standards, Certification, Neoliberalization

## Abstract

Sustainability standards have been one of the hopefuls for decades when it comes to ensuring the sustainability of biomass for the bioeconomy, especially in the wake of their evolvement from voluntary, non-governmental to hybrid, public–private governance instruments in recent years. In addition to doubts regarding their legitimacy and effectiveness, however, they have also been associated with a neoliberalization of nature that integrates natural resources into a free market logic. Drawing on a conceptual framework that builds on political ecology and the political sociology of policy instruments, this paper challenges this notion. To this end, it examines sustainability standards in three countries/regions particularly prominent for the bioeconomy—the EU, Brazil, and Indonesia—to illustrate how these can be differentiated in terms of their neoliberal orientation, and what can be inferred from this for the orientation and state of the respective bioeconomies. The results show that the introduction of sustainability standards is not necessarily accompanied by a neoliberalization of nature. Rather, it is shown that the standards and their specific designs—and thus also their intrinsic understanding of sustainability as integration—are primarily intended to serve the material interests of the state and the respective industrial factions, for which neoliberal configurations are sometimes seen as rather obstructive, sometimes as rather useful. The sustainability standards, and thus the bioeconomies for which they stand, therefore, rather serve as instruments to stay on the path of modernization and industrial development already taken or envisaged, or, put differently, as strategies to avoid social–ecological transformation.

## Introduction: the bioeconomy, sustainability, and standards

The political project of the bioeconomy (Goven and Pavone 2015) has already undergone several metamorphoses and variations since it first gained momentum in the EU and OECD starting in the mid-2000s (Patermann and Aguilar 2018; Vogelpohl and Töller 2021). In addition to monitoring and analyzing the global spread of bioeconomy policies and strategies in the last decade, one of the foci of social science research on the bioeconomy has therefore been the analysis and classification of different bioeconomy definitions, visions, and understandings (Böcher et al. 2020). Roughly, the relevant studies (e.g., Levidow et al. 2012; Bugge et al. 2016; Hausknost et al. 2017; Vivien et al. 2019) show a relatively consistent pattern of three competing understandings or visions of the bioeconomy: a biotechnology-oriented understanding that focuses on increasing the use and commercialization of biotechnological innovations, a biomass-oriented understanding that focuses on increasing the use and commercialization of all kinds of biomass-based products and processes, and an agro-ecological understanding that is closer to the vision of Georgescu-Roegens “bioeconomics” of an ecologically sustainable use of available resources and that subordinates economic growth to this goal (e.g., Georgescu-Roegen 1971; Mayumi 2001).

I do not aim to subscribe to or endorse one specific understanding or definition of the bioeconomy here. However, I will somewhat involuntarily focus on those bioeconomy visions that have become politically relevant and manifest in actual bioeconomy strategies and policies. Rather unsurprisingly, the three bioeconomy visions differ quite substantially in this regard: While the agro-ecological vision is virtually absent in them, the biotechnology- and the biomass-oriented visions both strongly influence actual bioeconomy strategies and seem to be about equally influential in this respect (e.g., Birch et al. 2014; Bennich and Belyazid 2017; Backhouse et al. 2017; Hausknost et al. 2017; Scordato et al. 2017; Vivien et al. 2019; Vogelpohl and Töller 2021).

This resonates well with the way these bioeconomy visions differ from one another in terms of their relation to the concept of sustainability (see Pfau et al. 2014; Priefer et al. 2017 for more on the relation between the two concepts in general). Both the biotechnology- and the biomass-oriented visions incorporate a form of weak sustainability, according to which natural resources can be replaced by human and physical capital and a system is sustainable as long as the total capital (consisting of natural resources, human and physical capital) remains the same or grows. The agro-ecological vision instead is equivalent to a form of strong sustainability, according to which natural resources form the basis for human and physical capital and are therefore not substitutable (Bennich and Belyazid 2017; D’Amato et al. 2017; Vivien et al. 2019).

Apparently, there is a clear correlation between the political relevance of the bioeconomy visions and the understandings of sustainability they represent. At the same time, the sustainability of the bioeconomy is seriously questioned, as the production of the necessary biomass still takes place under ecologically and socially detrimental conditions and has correspondingly harmful effects (e.g., Lühmann 2021). Thus, the sustainability of the bioeconomy needs to be governed, and in a way that is consistent with the prevailing vision of the bioeconomy, be it to actually make the bioeconomy more sustainable or to use sustainability as a “selling point,” which rather seems to be the case in political practice (Ramcilovic-Suominen and Pülzl 2018). Therefore, the instruments policy-makers choose to govern the sustainability of the bioeconomy are quite meaningful regarding the bioeconomy they envision.

There is a plethora of governance frameworks and potential policy instruments to choose from in this regard (e.g., Ladu and Blind 2017; Singh et al. 2021). One of them that is perpetually discussed in this context is sustainability standardization and certification of bio-based products since it is supposed to be able “to manage biogenic resources and their derived products in a sustainable manner” (Majer et al. 2018, p. 2, see also Bosch et al. 2015; Dietz et al. 2018, pp. 11–12). While there is no universally accepted sustainability standard for the overall bioeconomy, the instrument of standardization and certification has been one of the hopefuls for decades when it comes to ensuring the sustainability of biomass in different sectors of the bioeconomy. This has its origins primarily in private initiatives for the certification of wood-based products. The forerunner of this movement is the Forest Stewardship Council (FSC), which was founded in the 1990s as a multi-stakeholder initiative under the leadership of large NGOs such as the World Wildlife Fund (WWF) or Greenpeace, and exerted great influence on the forestry sector in the following years (e.g., Cashore et al. 2004). This model of private sustainability initiatives was soon followed by NGOs and companies in bioeconomy sectors such as coffee, palm oil, sugarcane or soy in particular and a number of initiatives emerged accordingly, particularly in the 1990s and 2000s (e.g., Marx 2012; Vogt 2019).

While the state initially played a mostly observational or advisory role in these market-based initiatives, this relationship changed from the 2000s on. A phase of hybridization began in which sustainability certification evolved from a voluntary, non-governmental to a hybrid, public–private policy instrument. Governance hybrids like this were hailed for combining the strengths of both private and public regulation, backing up the flexibility and innovativeness of the former with the legitimacy and authority of the latter (Cashore and Stone 2012; GIZ 2013; Gulbrandsen 2014). However, hybridization is not the only state response to private sustainability certification. From the 2010s on, both private and hybrid transnational schemes were increasingly met with resistance from state and industry actors in various world regions and bioeconomy sectors, who partly developed and instituted alternative sustainability certification schemes (see, e.g., Schouten and Bitzer 2015; Foley and Havice 2016; Vogelpohl 2021 for an overview). Thus, the instrument of sustainability certification has transformed considerably over the last decades. Nonetheless, it is still one of the main policy options for governing the sustainability of the bioeconomy, at least in certain sectors of it (e.g., Ugarte et al. 2020; Iriarte et al. 2021; Singh et al. 2021).

This paper aims to discuss what can be deduced from the adoption and design of sustainability standards about the orientation of current bioeconomies and the understanding of sustainability materializing in them. This endeavor is based on the political sociology approach to policy instruments, which postulates that policy instruments must be considered as carriers of general ideas about the role of the state in society and thus function as symbolic representatives of power and of specific ideas and views of the world (see, e.g., Lascoumes and Le Galès 2007; Kassim and Le Galès 2010). In this context, sustainability standards have often been classified as specific representatives of a “neoliberalization of nature,” as they would extend a competitive market logic to the field of natural resources (see, e.g., Heynen and Robbins 2005; Guthman 2007; Castree 2010b). Does the use of sustainability standards in a given bioeconomy thus represent its neoliberalization?

Against the background of the changing debate and use of sustainability standards in the context of the bioeconomy, this paper will challenge this very conclusion based on conceptual insights from the political sociology of policy instruments and the political ecology literature on the neoliberalization of nature. Subsequently, I will use the example of sustainability standards for biofuels in the EU and Brazil and for palm oil in Indonesia to illustrate how these can be differentiated in terms of their neoliberal orientation, and what can be derived from this for the orientation and state of the bioeconomies in the respective countries/regions. The aim of this paper is thus to investigate the meaning of specific sustainability standards for the regulation of the bioeconomy and to thereby provide a nuanced contribution to the contemporary debate on the “neoliberalization of nature.”

## Sustainability standards as tools of neoliberalization? Theoretical background and conceptual framework

### Standards as essentially political institutions

The sociology of policy instruments is based on the assumption that a policy instrument cannot be regarded as a merely technical, politically neutral problem-solving tool. Rather, it should be regarded as “a device that is both technical and social, that organizes specific social relations between the state and those it is addressed to” (Lascoumes and Le Galès 2007, p. 4). Thus, it always serves as a carrier and symbolic representative of general worldviews and ideas about the kind of state activity that is considered legitimate and effective. This approach tries to overcome the oft-encountered functionalist bias in policy instrument analysis, i.e., the focus on institutional structures and policy effectiveness that gives the impression of the choice of policy instruments as a predominantly technical endeavor, which “conceals what is at play politically” (Lascoumes and Le Galès 2007). Instead, it fundamentally builds on the premise that “policy instrumentation brings considerations of power to the forefront” (Kassim and Le Galès 2010, p. 5). Specifically, the depoliticization of fundamentally political issues as well as the masking of power relations through the allegedly technical nature of policy instruments are central power dynamics this approach seeks to unveil (Kassim and Le Galès 2010).

Voluntary standards are prime examples of these power dynamics. They are generally classified as a specific type of policy instrument that illustrates the neoliberal “tendency of state actors to delegate responsibility to private organizations” (Lascoumes and Le Galès 2007, p. 5). Thereby, they depoliticize the social conflicts they are supposed to resolve since they reduce “the discretionary nature of decision-making replacing it with a more ‘rules-based’ system over which civil servants and politicians have less active day-to-day control” (Kuzemko 2016, p. 109). Furthermore, although they are among the non-coercive policy instruments and thus give the impression of less invasive instruments, their combined scientific-technical and democratic legitimacy can exert strong coercion, sometimes almost having the force of law. This helps to mask underlying power relations, thus making them a highly political and a highly powerful policy instrument indeed (Lascoumes and Le Galès 2007, p. 14). This is even more true for hybrid standards that combine private and public regulation as they add an element of direct control and authority to the power dynamics at play.

Regarding the use and impact of policy instruments such as standards, Lascoumes and Le Galès therefore draw on James Scott's seminal book “Seeing like a state”, describing them in his words as "tools of legibility” (Scott 1998). In his book, Scott describes how modern states employ certain tools and techniques to make societies and citizens “legible” and thus governable from a top-down perspective. More specific to the research cases of this paper, Widengård et al. in their Scott-inspired paper titled “Seeing like a Standard” see most of these features in full swing when it comes to the hybrid EU biofuel sustainability standard. Accordingly, this standard provides EU policy makers as well as companies, NGOs and consumers with “a standard grid that makes biofuel plantations in far-away places legible” and their sustainability governable, while the authors also point to the fact that it is difficult, if not impossible, to define a uniform standard for sustainable biofuels (Widengård et al. 2018, p. 50). Thus, the conceptualization of policy instruments in general and of standards in particular through this lens as very context-specific, essentially political institutions seems particularly well suited for the purposes of this paper.

### Sustainability standards as neoliberalization of nature?

In political ecology literature, somewhat in line with the evaluation by Lascoumes and Le Galès, sustainability standards are often considered a representative policy instrument of a “neoliberalization of nature” (e.g., McCarthy and Prudham 2004; Heynen and Robbins 2005; Castree 2010b; Birch et al. 2010). The hallmark of the neoliberalization of nature is that the expansion of free markets into the environmental sphere is seen as a prerequisite for environmental protection (Beder 2001). It is thus a specific variant of the ecological modernization paradigm, whose linchpin is the general compatibility of capitalism with environmental protection. Within this “free market environmentalism” (Eckersley 1993), environmental problems are seen primarily as due to the faulty or lack of inclusion of environmental goods and services into the pricing of goods and services on the free market, i.e., as costs that are not internalized by their originators. To counteract this, proponents of neoliberal ecological modernization propose providing environmental goods and services with enforceable private property rights. The role of the state would then be to protect these property rights and ensure their enforceability.

With regard to the role of the state, neoliberalism is thus not necessarily characterized by deregulation—in contrast to laissez-faire liberalism, from which neoliberalism can be strongly distinguished in terms of its origins (e.g., Ptak 2016). Rather, the focus is on the privatization and/or commodification of (public) goods that were previously not subject to private property rights and/or were not marketed, as well as on the creation of competitive markets, the enforcement of which requires at least a selectively strong state. Concrete microeconomic policy measures derived from this are designed to stimulate trade, investment, innovation and competition, accompanied by market-friendly re-regulations or the promotion of market-flanking mechanisms such as voluntary standards and labels (Castree 2010a).

It is thus specific policy instruments that represent the trend toward such a neoliberal state that seeks not to impose a certain behavior but to encourage self-organization and that has increasingly displaced a more prescriptive, interventionist state since the 1980s (Braun and Giraud 2009). Accordingly, market-based policy instruments that characterize this neoliberal state have been on the rise since the early 1990s at the latest, especially in agricultural policy (e.g., Potter and Tilzey 2005; Dibden et al. 2009) and in environmental and climate policy (e.g., Parr 2012; Ciplet and Roberts 2017). As indicated in the previous subchapter, standards, labels and certification are instrumental representatives of this neoliberalization of nature. For example, Julie Guthman describes how food labels, far from representing resistance to such a neoliberalization of nature, are instead “an expression of roll-out neoliberalization”, as they “not only concede the market as the locus of regulation, but in keeping with neoliberalism's fetish of market mechanisms, they employ tools designed to create markets where none previously existed” (Guthman 2007, p. 456). Similarly, the introduction of sustainability standards for biofuels, embedded in the general rise of environmental and social standards especially in the agricultural and food sectors (Higgins et al. 2008), can be regarded an act of neoliberalization, since the negative effects of deregulated markets and global trade are to be combated here with the help of mechanisms that in turn create new markets (Vogelpohl 2015).

In this paper, however, I will put forward and substantiate the argument that a standard does not necessarily equal a neoliberal standard (see also Le Galès 2016), just as ecological modernization does not necessarily mean neoliberal ecological modernization, since it can be more neoliberal or more Keynesian. In the same vein, bioeconomy does not necessarily equal neoliberal bioeconomy. Kean Birch points out that while the bioeconomy transition pathway taken so far "might seem like a classic case of the neoliberalization of nature (…), a more complicated process is at play” (Birch 2021, p. 45, see also Birch 2019). It would therefore be an oversimplification to stamp the bioeconomy in general or the various bioeconomies in individual countries or regions with the label “neoliberal,” just as it would be overly simplistic to brand sustainability standards as neoliberal instruments across the board. Consequently, it would also be inadmissible to conclude that the use of sustainability standards in the context of a particular bioeconomy quasi automatically renders the latter neoliberal. Again in the words of Birch, it is rather the task "to unpack the manner in which policy tools and biophysical materialities configure bioeconomies in certain ways” (2021, p. 58), which this paper intends to do.

### Operationalization, case-study selection, and method

Against this background, this paper asks how neoliberal the sustainability standards used in the bioeconomy really are and what this tells us about the respective bioeconomies. More specifically, following the policy instruments approach (Lascoumes and Le Galès 2007; Kassim and Le Galès 2010) and the definition of neoliberalism (Castree 2010a) introduced in the previous subchapters, the following will be investigated in this paper:

- to what extent the standards create a competitive market for sustainability certification and sustainability certificates,
  - Is there a market for certification schemes on which obligated producers can choose between different, competing private sustainability schemes? Is there a market for the certificates on which they can be traded between investors and obligated producers?
- to what extent the criteria and processes defined in the standards (i.e., their understanding of sustainability) restrict or promote free trade of the biomass product in question,
  - How comprehensive are the criteria? Do they comprise social and ecological aspects? Are they designed in a way to comply with sustainability requirements in other jurisdictions? To what extent do they prevent the import of the biomass product in question?
- what the role of the state is both in instituting and in enforcing the standards in relation to the market and societal actors.
  - Who gets to decide on instrument choice in general and how it is designed in particular? What power dynamics and social relations both underlie the standards and are concealed by them? To what extent does this mask power relations and/or depoliticize the political nature of the issue?

These questions will be examined exemplarily with a view to three different bioeconomy-related sustainability standards to cover the varieties of sustainability standards in the context of the bioeconomy as well as their conditions and contradictions. The EU’s Renewable Energy Directive (RED) of 2009 is one of the most prominent and significant manifestations of transnational hybrid sustainability governance, since the sustainability of the biofuels used in the EU is to be proven via private, so-called voluntary certification schemes previously recognized by the EU Commission (e.g., Ponte and Daugbjerg 2015). The Indonesian Sustainable Palm Oil (ISPO) scheme is based on a state regulation that aims to certify all Indonesian palm oil production as sustainable by 2025 and was instituted at least partly as an alternative to transnational sustainability certification schemes. Another, albeit more RED-similar example is the relatively recent RenovaBio regulation adopted in Brazil in 2017 that ties Brazilian biofuel consumption to certain sustainability criteria, in particular the saving of a certain amount of greenhouse gas (GHG) emissions, with the aim of helping Brazil to reach its commitments under the Paris Agreement.

These are three similar but also different systems of using the instrument of biomass sustainability certification in the context of state regulation (see Table 1). Moreover, the three countries or regions are interesting case study countries, as they are important actors in the global bioeconomy in general, i.e., in terms of their salient role as producers, traders and consumers of bioenergy and its raw materials, as well as specifically in the biomass sustainability certification debate. Furthermore, they take different positions in the global bioeconomy, which are also reflected in the design of their respective sustainability standards.

**Table 1:** Overview of cases

Name (Country/Region)	Started	Structure	Sector addressed
RED (EU)	2009	Hybrid	Biofuels consumption
RenovaBio (Brazil)	2017	State-led	Biofuels consumption
ISPO (Indonesia)	2011	State-led	Palm oil production

Answering the above questions regarding these three cases will be embedded in brief outlines of their historical evolution and of their (supra-)national contexts. To this end, the cases have first been studied via secondary literature on the development of the particular biomass sectors within their respective broader societal contexts and political economies with a focus on the time frame from the 1990s onwards. These articles were initially searched in scientific search engines such as Google Scholar, SCOPUS, and Web of Science using certain keywords. Further articles were then found in a snowball sampling way via the articles cited in these articles. The same approach was used to find already existing secondary literature on the sustainability standards themselves. These were then assessed along the research questions outlined above through a selective content analysis based on the included research articles as well as on primary sources available online, i.e., material provided by the involved actors themselves (such as responsible governmental agencies, obligated producers, etc.) as well as civil society reports and statements and media coverage.

## Sustainability standards in the bioeconomy at work: case studies from the EU, Brazil and Indonesia

### RED in the EU: sustainability as a tool of reconciliatory neoliberalism

European bioenergy policy is deeply embedded in the bloc’s Common Agricultural Policy (CAP), which itself is historically situated between discourses of productivism, neoliberalism, and multifunctionality (Feindt 2018). It thus represents the more or less continuous neoliberalization of the EU as a whole since the 1980s (van Apeldoorn 2002). The EU’s biofuels policy and especially the sustainability standard built into it can be read in this context as an example of neo-productivism, i.e., an attempt to secure public funding for agriculture in the wake of the neoliberalization of the CAP and the corresponding reduction of public support for food production in the 1990s (Ward et al. 2008; Wilson and Burton 2015).

In the 2000s, biofuels have been regarded as an economically and ecologically viable alternative to gasoline and diesel in the transport sector by many policy-makers and industry representatives in Europe. For them, biofuels seemed to promise less dependency on crude oil imports, a reduction of carbon emissions and prospects for European agriculture, even though all these assumptions were never undisputed (Londo and Deurwaarder 2007; Franco et al. 2010; Leopold 2010; Pacini et al. 2013). Eventually, in 2008, the European Commission (EC) proposed a binding 10% target for biofuels to be met by all Member States in 2020. Out of a need to react to the by then already heavily voiced social–ecological problems arising from such an increased biofuels usage, the EC proposed tying this target of the compliance of the respective biofuels with certain sustainability criteria (e.g., Franco et al. 2010; Pilgrim and Harvey 2010; Leopold 2010; Vogelpohl 2015). The RED eventually determined several sustainability criteria to be fulfilled by the biofuel producers (reduction of greenhouse gas emissions; no raw material from land with high biodiversity value; no raw material from land with high carbon stocks), thus covering only the environmental dimension of sustainability (European Union 2009, pp. 36–37).

The predominant option for biofuel producers offering their product on European markets to demonstrate compliance with these criteria is certification via EC-recognized private sustainability schemes. This means that a private scheme needs to be successfully assessed by the EC against the coverage of sustainability criteria laid down in the RED as well as against the requirements regarding documentation management, independent auditing and a mass balance system (European Commission 2010). With the 2015 ILUC (indirect land-use change) directive, these requirements have been extended, now also comprising aspects of transparency, internal monitoring and conflict resolution (European Union 2015). Thus, assessment and recognition are restricted to the mandatory criteria of the RED and some procedural aspects, while nonmandatory sustainability aspects like social issues are not assessed during this process (European Court of Auditors 2016). The approach to ensuring the sustainability of bioenergy consumed in the EU via standards and certification essentially has not changed since it was first adopted in 2009, even though it has been widely criticized for being ineffective and unjust (e.g., European Court of Auditors 2016; Widengård et al. 2018; Vogelpohl and Perbandt 2019).

However, the changes that have been made show that its main function was and still is a conciliatory one. Two episodes from the 2010s are particularly illustrative of this. The first one concerns the (non-)integration of ILUC into the standard’s criteria. The EU commission procrastinated the ILUC dilemma in 2009, but had to deal with it in the following years. After years of fierce debate between NGOs, who had made ILUC their prime strategy for overthrowing EU biofuels policy as a whole, and the biofuels industry, it was finally decided to not integrate ILUC, but to rather put a cap on biofuels of the 1st generation (Levidow 2013; Hübner 2014). The procrastination of the issue and the shifting of the discourse arena from a public to a more undisclosed, scientific-technical one also led to “the systematic ‘closing down’ of broader political debates around the issue,” i.e., to its depoliticization, much to the frustration of thereby disempowered NGOs (Palmer 2012, p. 495, see also Levidow 2013).

The second one concerns the singling out of palm oil when it comes to sustainable biofuels in the recast of the RED in 2018 (RED II), according to which the use of so-called “high ILUC-risk biofuels”, such as palm oil-based biodiesel, is to be capped at 2019 consumption levels until 2023 and then phased out until 2030 (European Union 2018). This does not technically mean an import ban on these biofuels, but severely hampers their attractiveness in the EU market, since these biofuels will not be eligible to count towards the target set up in the directive. In a concretization of this “freeze and phase-out” regulation by the EC (European Commission 2019), the methodology for determining such high ILUC-risk biofuels was defined in a way that only palm oil qualifies as a high ILUC-risk feedstock. Consequently, palm-oil-based biofuels are the only biofuels covered by the freeze and phase-out regulation (Tyson and Meganingtyas 2020; Mayr et al. 2021).

In terms of the neoliberal character of this EU bioenergy sustainability regulation, on the one hand, the scope and ambition of the criteria is clearly subordinate to the premises of free trade since “the EU criteria were developed with WTO rules expressly in mind” (Kay and Ackrill 2012, p. 302). This applies both to the precise formulation of the ecological criteria and to the omission of social criteria. The latter were deliberately neglected with a view to WTO criteria, as it was feared that they would collide with WTO rules and, therefore, “would overstep some countries’ ‘red lines’ and thus would almost certainly trigger an action in the WTO” (Ackrill and Kay 2011, p. 560). On the other hand, the introduction of the sustainability standard, at least in part, also is a strategy to protect domestic agriculture, as can be seen, for example, in the singling out of palm oil under the EU RED II.

The sustainability regulation for bioenergy under the RED is thus a prime example of EU agricultural policy caught between neoliberalism and productivism (Feindt 2018). This means that a potential instrument has to fit with both paradigms. Standards and certification do just that since they restrict and facilitate trade with bioenergy products at the same time. On the one hand, European producers are protected from external competition to a certain degree. On the other hand, it does not interfere with global trade too much and European importers can use these standards to shape trade conditions with exporters (Staricco and Buraschi 2022). Moreover, as Vogelpohl notes, the introduction of the EU sustainability criteria can be embedded and contextualized in the overall European integration project (Vogelpohl 2015). In this case, sustainability criteria represent the compromise between the two factions of European capital, the national champions and the global competitors, and their representations in the respective state apparatuses (van Apeldoorn 2002).

Furthermore, while it is not an inherently neoliberal instrument, it is also a prime example of a market-flanking mechanism in Castree’s sense in the context of an overarching, market-creating policy strategy for biofuels. Apart from the fact that the EU sustainability regulation does not create the market for biofuels itself (the binding target does), it creates another market, namely that for certification schemes. Thus, thanks to EU regulation, a global certification market has developed where private, corporate schemes compete with multi-stakeholder initiatives for biomass sustainability certification for the EU biofuels market (Henriksen 2015).

Thus, the state plays a pivotal role in EU bioenergy sustainability policy. As noted by many scholars, the biofuels sector is politically instituted and simply would not exist without massive state intervention (e.g., Pilgrim and Harvey 2010). That, however, does not mean that the state is a monolithic power bloc that has unambiguous convictions and interests when it comes to biofuels. Rather, as Brand et al. note, it has to be seen as “a strategic yet asymmetric terrain on which competing and antagonistic interests of social forces are being organized, articulated and translated into specific policies” (Brand et al. 2022, p. 284). Thus, it is these competing forces within the state that form the background against which the RED sustainability standard for biofuels takes on a reconciliatory role. The introduction of sustainability certification into the RED always was an attempt of reconciling the neoliberal with the productivist version of the European biofuels project, in which the state acts both as the market creator and the market protector to a certain degree. Thus, it also is a prime example of the dominant interpretation of sustainable development as “a non-adversarial approach to environmental politics” (Hajer and Fischer 1999, p. 4), which is representative not only of European biofuels policy but arguably of the European bioeconomy as a whole (e.g., Ramcilovic-Suominen and Pülzl 2018; Vogelpohl et al. 2021).

### RenovaBio in Brazil: sustainability as a tool of authoritarian neoliberalism

Both neoliberalism and authoritarianism are deeply embedded within Brazil’s political economy (Søndergaard 2021). After an era of re-democratization—or, a more embedded neoliberalism—following the military dictatorship from the 1980s onwards, in which political processes became more inclusive, especially during the Lula/Rousseff era (2003–2016), the short presidency of Michel Temer (2016–2018) marked the return to a more authoritarian—or, a more disembedded—neoliberalism (da Silva and Veiga Vieira Mancio Bandeira 2021). And it is exactly this period in which the RenovaBio policy was adopted.

After the oil price crisis of 1973, supporting biofuels—especially sugarcane-based ethanol—was one of the strategies of the Brazilian state to secure national energy supply. Therefore, the Brazilian military dictatorship under the presidency of Ernesto Geisel in power at the time launched the Proálcool Program in 1975 (Mingo and Khanna 2014). This sparked the development of a sizable ethanol industry in Brazil through the 1970s and 1980s and laid the foundation for today’s relatively big role of ethanol in the Brazilian transport sector at a share of about 20% (Lima and Fabiano 2020; Bastos Lima 2021).

After lower oil prices disincentivized the use of ethanol during the 1980s and 1990s, a biofuel resurgence took place under the Lula administration from 2003 to 2010, which was heavily aided by the development and market establishment of flex-fuel cars that could run on any mixture of gasoline and ethanol. Furthermore, the Lula phase of biofuels policy in Brazil was characterized by the attempt to establish a biodiesel industry with the explicit goal of integrating smallholders into the value chain. The 2006 agroenergy plan integrated these approaches and combined biofuels support with sustainability measures such as the social fuel seal or agro-ecological zoning (Stattman et al. 2013; Bastos Lima 2021). After the Lula phase, the 2015 Paris Agreement and the respective Nationally Determined Contributions (NDCs; GHG reductions of 37% and 43% from 2005 levels by 2025 and 2030 for Brazil) ware catalysts of new policy action in the field of Brazil biofuels policy. At the same time, Brazil politics were in a state of severe political turmoil after the dismissal of Dilma Rousseff and the takeover by Michel Temer in 2016 (Søndergaard 2021).

Against this backdrop, the RenovaBio policy was presented to the Brazilian Parliament by the Biodiesel Parliamentary Front leader in November 2017, and adopted very quickly only two weeks later (Takaes Santos 2020). Through RenovaBio, the National Council for Energy Policy (CNPE), which is chaired by the Ministry for Mining and Energy (MME), derives yearly GHG emission reduction targets for obligated fuel suppliers directly from Brazil’s NDCs. The program provides the framework to certify the efficiency of biofuel production regarding the reduction of GHG emissions based on a life cycle assessment that assigns a specific carbon intensity to each unit of biofuel. The difference between this and the default value for fossil fuel carbon intensity indicates the amount of avoided GHG emissions per unit. For one ton of avoided GHG emissions, biofuel producers or importers get one so-called CBIO, i.e., the currency of decarbonization credits. These CBIOs are issued by contracted inspection companies (that are accredited to carry out the biofuel certification by the National Agency of Petroleum, Natural Gas and Biofuels—ANP) and can then be traded by the producers/importers on the Brazilian stock exchange (B3). There, they can be purchased and resold by financial investors before they have to be withdrawn from the market by obligated fuel suppliers for them to comply with the stipulated yearly reduction targets (e.g., Martinelli et al. 2022).

Initial reduction targets had to be reduced shortly after RenovaBio started operating in December 2019 in the wake of the Covid-19 pandemic that broke out just a few months later and contorted the Brazilian fuel market. Consequently, the Brazilian government reviewed RenovaBio’s targets and cut the 2020 target by 50% (from 29 million to 14.53 million CBIOs, of which eventually 97.6% were retired by obligated fuel suppliers in 2020). The MME and CNPE also proposed reduced targets for the entire period, with the difference between the original and adjusted 2030 plans ultimately amounting to only 10%, which still represents a significant increase in biofuel use over that time span (Grangeia et al. 2022).

Besides this calculation, biofuel production under the RenovaBio scheme has to comply with further eligibility requirements that are supposed to link biomass production to sustainable land use. Thus, biomass production for eligible biofuels is disallowed on land that was native before November 2018 to avoid further deforestation and has to comply with local environmental legislation (like the Rural Environmental Cadaster—CAR) and agro-ecological zoning (Morandi 2020). However, agro-ecological zoning for sugarcane, a regulation that identifies areas where cultivation of sugarcane should be sanctioned and where it should be prohibited, was abolished by the new right-wing President Bolsonaro (who kept RenovaBio largely intact otherwise) in November 2019 so that only the first two criteria are left (Andrade Junior et al. 2020).

Regarding only the instrument of RenovaBio itself, it resembles a textbook example of neoliberal environmental policy. Its proponents literally describe it as “a market driven incentive mechanism, based on the economic theory of ‘Coase Market’” (Morandi 2020), in other words, a market in which externalities are priced in advance, thus minimizing transaction costs and optimizing efficiency. GHG savings are quantified, privatized and marketed transnationally on the stock market. In addition, the entry barriers to this market are very manageable according to the few criteria that must be met in addition to GHG savings. Unlike RED, however, RenovaBio does not use existing (or even yet to be established) private certification systems to prove compliance, even though it is currently debated how private schemes such as the sugarcane-focused Bonsucro scheme could be integrated into RenovaBio certification (e.g., Bonsucro 2020, p. 21).

The state plays a differentiated role here. On the one hand, it is largely on the outside of the market it has created and, in this sense, a neoliberal state in the best sense. On the other hand, it is closely linked to the sugarcane industry and, in various forms, directly involved in the biofuel business. This also applies to the policy formulation process on RenovaBio, in which representatives from the involved industries were heavily involved and able to shape the policy, while non-governmental organizations (NGOs), community representatives or smallholders were largely marginalized, which clearly shows the asymmetric power relations between these actors in Brazil (Lazaro and Thomaz 2021). Supporting this finding, Takaes Santos finds that a lack of participation of NGOs was notable, suggesting a dominance of experts and the private sector in the policy process. This is especially true for the Brazilian sugarcane association UNICA, whose current president, Evandro Gussi, also is the leader of the Biodiesel Parliamentary Front that introduced RenovaBio to the Brazilian Parliament (Takaes Santos 2020).

In line with that, although on a more general level, Bastos Lima identifies two broad actor coalitions in Brazilian biofuels governance: an agribusiness and an agro-ecology coalition, of which the former is dominant (Bastos Lima 2021, pp. 109–111). Aamodt sees a similarly dominant actor coalition in Brazilian energy policy in general that forms an iron triangle of energy policy actors that make policy decisions in the CNPE, not in the Congress (Aamodt 2018). In the case of RenovaBio, these actors gather around narratives of sustainability as energetically and environmentally efficient economic development based on technological innovation, which speaks to “the dominance of a clear ecological modernization discourse in the RenovaBio’s policy formation and implementation” (Lima and Fabiano 2020, p. 8).

Circling back to the beginning of this section, RenovaBio and the way it is designed and implemented thus bears witness to the way a dominant actor coalition tries to fit Brazil's bioeconomy into the overarching paradigm of authoritarian neoliberalism that is on the rise again in Brazil ever since the Temer administration. The sustainability standard that RenovaBio is based on—just as the closely intertwined environmental cadaster CAR—therefore is a specific instrumental representation of this worldview and the way it materializes in the Brazilian bioeconomy (see also Siegel et al. 2022).

### ISPO in Indonesia: sustainability as a tool of national sovereignty

Indonesia is the biggest palm oil producer and exporter in the world and responsible for more than 50% of the world palm oil production. Accordingly, the palm oil industry plays an important role in Indonesian politics, society and economy (e.g., Cramb and McCarthy 2016; Bastos Lima 2021). It is rooted in the nineteenth century when Dutch colonialists brought oil palm trees to Indonesia. Roughly a century ago, the colonial Dutch East Indies became one of the major global producers of palm oil. After the independence of Indonesia in 1945, however, palm oil lost its paramount status for the Indonesian economy. It had a renaissance as a national commodity under the Suharto regime that took power in Indonesia in 1966 and first ramped up public investment in palm oil plantations and then, aided by the IMF, channeled private investments into the sector (Choiruzzad et al. 2021).

The real boost for the Indonesian palm oil sector, however, only came with the fall of the Suharto regime in 1998 and the subsequent democratization. Choiruzzad et al. (2021) identify three reasons for this: liberalization (i.e., foreign investments), state decentralization, and rising international demand led to Indonesia’s current dominant position in the global palm oil sector. This, however, came not only with a lot of local corruption and mismanagement, but also with severe environmental destruction. As of the 2000s, this aspect rose to the public fore and especially international NGOs from palm oil-importing countries started to campaign against the practices of the Indonesian palm oil industry. This culminated in the foundation of the Roundtable for Sustainable Palm Oil (RSPO) in 2004, a multi-stakeholder roundtable initiated in Switzerland by the WWF together with corporations based in the Global North that ever since “has been a site of contention between social forces with different interests and concerns” in Indonesia (Choiruzzad et al. 2021, p. 198, see also Sinaga 2022).

Thus, the sustainability of palm oil became a major concern for the Indonesian palm oil-industrial complex with the Indonesian state’s attitude towards the RSPO developing from indifferent via cooperative to antagonistic, which ultimately led to the initiation of the ISPO in 2011 (Wijaya and Glasbergen 2016). It was started and spearheaded by the Indonesian state and the ISPO sustainability principles and criteria are strongly aligned with existing legal and regulatory requirements, which is why it is sometimes referred to as Indonesia’s “legality standard” for palm oil (Hospes 2014). This implies that the ISPO is much broader than, for example, the RED or the RenovaBio standard, as it includes social sustainability criteria in addition to environmental ones, such as labor conditions, social responsibility and community empowerment. The ISPO very much resembles the RSPO in this regard. With regard to the actual level of ambition and performance, however, studies have concluded that the RSPO as well as most other comparable schemes tend to perform better, despite considerable potential for improvement on their part as well (e.g., Hospes 2014; efeca 2015; Pirard et al. 2017; Kusumaningtyas 2018).^1^

While the ISPO was initially meant to be mandatory for all palm oil production in Indonesia, it was revised in 2015 to the extent that greater differentiation was now made between the various palm oil producers and uses. Thus, the standard remained mandatory for plantation companies, while smallholders or plasma farmers as well as production for the bioenergy market remained exempted (Astari and Lovett 2019; Glasbergen 2018). The ISPO was revised again in 2020, when the Indonesian government escalated the legal status of the ISPO from a Ministry of Agriculture Regulation into a Presidential Regulation that stipulated that all Indonesian palm oil production is to be ISPO-certified by 2025. This applied immediately to plantation companies, while smallholders are allowed a 5-year transition period before ISPO compliance will be enforced (Choiruzzad et al. 2021). Additionally, the ISPO principles and criteria were revised slightly (a transparency principle was added while the principle on the protection of primary forests and peatlands was removed). In the eyes of civil society observers, however, this ISPO reform “falls far short of meeting expectations and ensuring palm oil in Indonesia is sustainable” (Kaoem Telapak and Environmental Investigation Agency 2020, p. 2).^2^

Looking at the ISPO through the conceptual lens of this paper, the ISPO is by no means a neoliberalizing instrument in that it privatizes or marketizes something that was not privatized or marketized before. Rather, it regulates the already existing market for palm oil in Indonesia. Furthermore, the ISPO also creates a market neither for sustainability certificates nor for sustainability certification schemes. The certificates are not tradeable (such as in Brazil) and there are no private certification schemes competing in a certification market (such as in the EU; although ISPO is, as is RenovaBio, relying on independent, private certification bodies to perform the certification). Palm oil producers (be it corporations or smallholders) cannot choose to get certified, e.g., to realize a price premium on the market, but are forced to do so via regulation.

Regarding the sustainability criteria, the ISPO standard is rather diversified in comparison to the RED or RenovaBio. It comprises a broader range of principles, including social ones, even though the implementation of the ISPO remains rather weak so far (see, e.g., Hidayat et al. 2018; Choiruzzad et al. 2021; Putri et al. 2022). In terms of global trade, however, the broad scope of the criteria is not supposed to be an obstacle, but rather quite the opposite. In fact, one of its explicit goals is to “increase the acceptance of Indonesian palm oil in the global market” (Astari and Lovett 2019, p. 4) as it seeks “to protect the palm oil industry from external pressures” (Choiruzzad et al. 2021, p. 204) and “to open up new markets for certified palm oil beyond the European one” (Vogelpohl 2021, p. 8, see also Wijaya and Glasbergen 2016). For that to work, however, the governance and implementation of the ISPO would have to be improved considerably “to gain better credibility on sustainability abroad” (Putri et al. 2022, p. 1).

The Indonesian state takes a pivotal position both in the adoption and the implementation of the ISPO. This is, however, not to say that it is a monolithic bloc in this context. Choiruzzad et al. place the conflict around the certification of palm oil and the establishment of the ISPO in the context of processes of fragmentation and internationalization of the state, as to which “not all government units automatically side with the palm oil industry” (2021, p. 200). Thus, the ISPO is the result of a complex process of compromise between different social forces that are all reflected in the state apparatus. At the center of this process is the palm oil–industrial complex, which Pye—in terms of Indonesia—describes as the close connection of the state with the Indonesian palm oil industry (Pye 2016, see also Kapriadi 2019; Choiruzzad 2019). Thus, the centrality of the role of the Indonesian state is not to say that the palm oil industry does not play an important role in the development of the ISPO. Rather, it means that, “at the same time, reformists within the state bureaucracy internalize global norms of sustainability and attempt to push reforms in the governance of the palm oil industry” (Choiruzzad et al. 2021, p. 204). In line with this, Astari and Lovett in their discourse analysis of Indonesian palm oil politics find “diverse and conflicting beliefs amongst the stakeholder groups promoting sustainability in the palm oil sector” (Astari and Lovett 2019, p. 4).

As a result of these internal struggles, the ISPO is an ambiguous policy that is at least as much inward-looking as it is outward-looking and that serves to integrate several currents within the Indonesian state and society. At the same time, however, there is a common theme under which the ISPO sustainability standard and the politics surrounding it can be subsumed, and that is not neoliberalism, but sovereignty. Thus, also Astari and Lovett’s “main findings confirm that ISPO initiation was triggered by a need for sovereignty” (Astari and Lovett 2019, p. 2). This is in line with other findings that see the initiation of the ISPO sustainability standard as characterized by a specific interpretation and discursive linkage of sovereignty and sustainability (Vogelpohl 2021, see also Hospes 2014; Schouten and Hospes 2018; Higgins and Richards 2019; Hinkes 2019; Sinaga 2022) that very much construe palm oil as an inherently sustainable national commodity, whose promotion and protection is the sovereign function of the Indonesian state and its bioeconomy.

## Summary and conclusions

The three sustainability standards examined in this article show some variety in terms of their neoliberal orientation (see Table 2). These relate both to the scope of the criteria to be complied with and to the way in which this compliance is to be demonstrated. While the list of criteria of the Indonesian system ISPO is relatively comprehensive (at least on paper), the European RED and the Brazilian RenovaBio are limited to (relatively few) ecological criteria. Similarly, the cases differ in terms of market creation and design. In this context, RenovaBio is—as far as the instrument itself is concerned—an almost textbook example of a neoliberal environmental policy, since it artificially creates an allowance market on which free trade is then supposed to lead to efficient distribution. The RED also creates a market, but rather a market for certification schemes, not for certificates. In the case of the ISPO, no market is created at all, but rather the already existing market is regulated.

**Table 2:** Summary of results

	Market creation	Relation to free trade	Role of the state
RED	Creates the market for biofuels and for certification schemes (not for certificates)	Sustainability generally subordinate to free trade, but selectively protectionist	Reconciliatory creator of the market for biofuels and protector of domestic industry
RenovaBio	Creates the market for biofuels and certificates (not for certification schemes)	Sustainability a means of promoting free trade (of biofuels and certificates)	Neoliberal creator of the market for biofuels and protector of domestic industry
ISPO	Rather regulates than creates the market for palm oil (no markets for certificates or certification schemes)	Sustainability not hindering, but primarily preserving access to foreign palm oil outlets	“Sovereign” regulator of the market for palm oil and protector of domestic industry

In terms of their relation to free trade, none of the standards generally aim at hindering global biomass trade. The EU RED, however, despite its general subordination to WTO rules, has a certain protectionism to it since it is at least partly used to shield the EU market for vegetable oil from too much influx of foreign (esp. Indonesian and Malaysian) palm oil. RenovaBio, in contrast, is designed not only to create a domestic GHG market, but specifically to open this market to investors and thus attract foreign capital. The ISPO, finally, serves as a tool to at least sustain and at best improve access to the global market for Indonesian palm oil and thus protect the palm oil industry. What can be seen here is that the relation of the standards to trade in all three cases is an instrumental one, not an ideological one, meaning that trade relations are supposed to be used in the way that best fits the respective domestic industries. Always depending on whether marketization and deregulation or regulation and protection are seen as better means to this end, sustainability standards will be designed accordingly.

The role of the state, finally, is a pivotal one in all three of the sustainability standards. This is not to say, however, that it is not a neoliberal one. As explained in the conceptual section, the neoliberal state is not a weak, passive state, but a selectively strong one that creates and enforces competitive markets. With regard to the sustainability standards regarded here, this selectively strong state can be seen first and foremost in the Brazilian RenovaBio standard and in the European RED. In both cases, the sustainability standards are almost textbook examples of market-flanking mechanisms that accompany the state-instituted markets for biofuels that are created in the frameworks of the same policies. The RenovaBio standard can thus be described as quintessentially neoliberal, both in terms of its design and its function, whereas the RED standard is so regarding the function only. Regarding the ISPO, in contrast, the state does play a central role, but not in a neoliberal way, but rather in a neo-mercantilist and sovereignty-claiming way that aims at governing and controlling the domestic palm oil sector and its position on the global market.

Against this backdrop, the answer to the question of whether the introduction of these sustainability standards is accompanied by a neoliberalization of the respective bioeconomies must be differentiated and read: not necessarily. The neoliberal orientation of sustainability standards is not a feature inscribed in the instrument per se. They do not necessarily valorize nature, endow it with private property rights, and commodify it. Instead, they can also be more interventionist in nature, as the example of the ISPO standard shows.

What applies equally to all three standards considered here, however, is that the state and the involved industrial factions are the dominant centers of power and that the introduction and design of the standards are intended to serve their respective material interests, for which neoliberal configurations are sometimes seen as rather obstructive, sometimes as rather useful. In all three cases, sustainability standards have an internal and an external dimension in this respect. In the case of the EU, in addition to market creation, the protection of domestic biofuel producers or the corresponding raw material producers plays an important role in the introduction of the RED standard, as exemplified by the singling out of palm oil under the RED II. Therewith, the EU also exercises extraterritorial control over natural resources in other regions of the world (e.g., Bastos Lima and Gupta 2014), to which the ISPO, in turn, is a direct, rather neo-mercantilist reaction by the Indonesian palm oil-industrial complex. Externally, the ISPO is thus intended to secure Indonesian palm oil's access to world markets, to the EU market in particular, as well as to improve its international credibility on these markets. Internally, the ISPO rather addresses intra-Indonesian competition for control over domestic palm oil governance. The image gain on the international level as well as the development of corresponding transnational markets—in addition to obligations under the Paris Agreement—also represents the external dimension in the case of RenovaBio, whereas the internal dimension in the form of boosting domestic biofuel demand and the corresponding support of the sugarcane industry was arguably more relevant in this case. Nonetheless, the direct interrelations between the EU RED and the Indonesian ISPO as well as the transnational prospects of RenovaBio point to the global entanglements and interdependencies of such policies and markets for sustainable biomass, which deserve further academic attention.

Consequently, paraphrasing Widengård et al. (2018), “seeing like a sustainability standard” does not mean that every standard sees the same sustainability. Rather, what sustainability it sees depends on the specific design of this standard and on who created it and with what interest. Sustainability in this context does not necessarily look like private property rights and free markets, but can also look like sovereignty and state intervention. Thus, circling back to aspects brought up in the conceptual part of this paper, the instrumentation approach, i.e., looking at the specific instruments of bioeconomy policy and their designs, does offer a maybe not exhaustive, but unique perspective on the politics of the bioeconomy and “the importance of the power dimensions that underlie the choice of instruments” (Kassim and Le Galès 2010). The standards adopted in these three cases can indeed be considered manifestations of general worldviews and ideas about what is deemed legitimate and sustainable in the respective bioeconomies, both in terms of the processes of their adoption and in terms of their functions and effects. In this context, the assessment of Lascoumes and Le Gales that standards rely “on a mixed legitimacy that combines a scientific and technical rationality, helping to neutralize their political significance” (Lascoumes and Le Galès 2007, p. 14), i.e., helping to depoliticize them, can at least generally be confirmed.

However, this paper also shows that there is no standardized standard in this sense. For example, the three standards considered here cannot be uniformly regarded as “tools of legibility,” as Scott put it (1998), since this aspect of the standards is far more pronounced in ISPO and RED standards than in the RenovaBio one. And it has also become clear that the sustainability standardization of different bioeconomies does not necessarily lead to their uniform neoliberalization, if at all. Thus, sustainability standards (as all standards and all instruments even) are what is being made of them—and not inherently neoliberalizing, as this paper has shown (see also Le Galès 2016), or even sustainability-promoting (e.g., Staricco and Buraschi 2022). In fact, what this paper has also shown with regards to the latter is that this is hardly the case with the three standards considered here since they all understand sustainability in the sense of a sustainable development as integration discourse (Hugé et al. 2013), i.e., in the non-adversarial sense of ecological modernization. In all three cases, therefore, the sustainability standards, and thus the bioeconomies for which they stand, rather serve as instruments to stay on the path of modernization and industrial development already taken or envisaged in the respective political economies, or, put differently, as a strategy to avoid social–ecological transformation.

## Footnotes

1 In a report from the Forest Peoples Programme ranking certification schemes for biofuels and edible oils, for example, ISPO was the lowest-ranked scheme, even lower than the similar state-led Malaysian Sustainable Palm Oil (MSPO) scheme (McInnes 2017).

2 Especially in conjunction with the Job Creation Act that was adopted later in 2020, which all but abolishes the need for impact assessments or community involvement in processes of environmental planning or designating forest and, therefore, severely weakens ISPO, as it is largely based on the laws and regulations in Indonesia that are severely weakened by the new bill (Kaoem Telapak and Environmental Investigation Agency 2020, p. 5).
